# Cost of Hospital Treatment Claimed from a Doctor

**Published:** 1914-05-30

**Authors:** 


### Cost of Hospital Treatment Claimed from a Doctor.

Dr. Denarié, of Chambéry, once sent a "pauper" of that town with a medical certificate to facilitate his reception into the Hôtel-Dieu of Lyons. After being examined by the interne on duty at that hospital the patient was admitted by the "bureau des entrées," probably as the result of an oversight, as he had no authority from the Mayor of Chambéry guaranteeing the taking over of the expense of treatment.

Then this extraordinary thing happened: the Administration of Hospitals of Lyons, unable to obtain payment of these expenses from the insolvent patient, and the municipality of Chambéry also refusing to pay up, claimed them . . from Dr. Denarié! In the course of a letter to the doctor, the municipality stated: "The commission for the settlement of disputed points of my Administration called to examine this situation estimated that the cost of treatment of the patient X could not legally be charged to the hospitals of Lyons, and that, in default of repayment by the family or by the town of Chambéry, it rests with the doctor who gave orders for the transfer of the patient to the Hôtel-Dieu to stand surety for the payment of the expenses. Consequently, I have the honour to ask you, M. le Docteur, to be so good as to use your influence so that the cost of treatment of Mr. X, whom you have sent to our hospitals, shall be repaid to my Administration by the family of the patient or by the town of Chambéry. In default of this, it will be your duty to settle the account yourself. The sum due for eighty-two days at five francs amounts to 410 francs. I must let you know that the cost per day for patients treated in our surgical wards at the expense of the family or of private individuals is five francs. This cost amounts to 3 fr. 85 in cases when the expenses of hospital treatment are taken charge of by the communes." "To ask a doctor for 410 francs because his signature figures at the bottom of a strictly medical certificate intended merely to facilitate examination," remarked *La Province Médicale* at the time, "and because an employee of the aforesaid Administration has committed the blunder of not demanding from the patient the documents necessary for his admission, really passes all understanding." And the Concours Médical pointed out to Dr. Denarié the line of conduct he should take up by advising him "purely and simply to send the Administration of the civil hospitals of Lyons about its business, and not to be troubled by threats which this Administration knows well enough have no legal weight whatever."

